# Differences in Prevalence of Pregnancy Complications and Placental Pathology by Race and Ethnicity in a New York Community Hospital

**DOI:** 10.1001/jamanetworkopen.2022.10719

**Published:** 2022-05-05

**Authors:** Peilin Zhang, Sylvia Dygulski, Farah Al-Sayyed, Beata Dygulska, Sanford Lederman

**Affiliations:** 1 Department of Pathology, Weill Cornell Medicine, New York Presbyterian-Brooklyn Methodist Hospital Brooklyn, New York; 2 Department of Pediatrics, Weill Cornell Medicine, New York Presbyterian-Brooklyn Methodist Hospital Brooklyn, New York; 3 Department of Obstetrics and Gynecology, Weill Cornell Medicine, New York Presbyterian-Brooklyn Methodist Hospital Brooklyn, New York

## Abstract

This case series study compares the prevalence of common pregnancy complications and placental pathologies by race and ethnicity among individuals treated at a community hospital in New York City.

## Introduction

The association of maternal race and/or ethnicity with pregnancy complications and neonatal morbidity or mortality has been extensively documented. Over the previous 2 decades, non-Hispanic Black and Hispanic women in pregnancy have had a higher incidence of gestational diabetes, preeclampsia and pregnancy-induced hypertension, preterm birth, and stillbirth.^1,2^ Racial and ethnic disparities in outcomes for placental pathology have been less studied. Even in a large cohort study of placental pathology, only non-Hispanic Black and non-Hispanic White pregnant women were compared and not all racial minority groups were included.^3,4^ We sought to examine the association of race and/or ethnicity with placental pathology as well as pregnancy complications at a community hospital in New York City.

## Methods

This case series study included all singleton placentas in the third trimester submitted for pathology examination in March 2020 and November 2021 with the exception of twin or multiple births. Placental examination at New York-Presbyterian Brooklyn Methodist Hospital is criteria-based and performed according to the standard procedure (eMethods in the Supplement).^5^ Placental pathology data, neonatal birth data, and maternal racial and ethnic data were retrieved from medical records based on standard national criteria. Information on gender was also gathered from medical records—all pregnant individuals in the data population were women. Classification for race and ethnicity included Asian, Hispanic, non-Hispanic Black, and non-Hispanic White categories; responses outside of these categories (ie, “unknown,” “others,” or “declined”) were recorded together as 1 group. Laboratory tests of white blood cell counts with differentials and blood pressure measurements were from preadmission tests for delivery only. Statistical analysis was performed by using Rstudio version 1.3 (R Project for Statistical Computing) including baseline characteristic table and multivariant ANOVA tests. *P* < .05 was considered significant in 2-sided tests. Institutional review board approval was not required according to section 46.101(b) of US Department of Health and Human Services regulation 45CFR 46, and this study was exempted from informed consent requirements because data were deidentified. This study followed the Strengthening the Reporting of Observational Studies in Epidemiology (STROBE) reporting guideline.

## Results

A total 3724 singleton placenta with clinical, neonatal birth data and maternal racial data were available for study, including 155 Asian (4.2%), 1291 non-Hispanic Black (34.7%), 315 Hispanic (8.5%), and 1662 non-Hispanic White mothers (44.6%), and 301 patients (8.1%) with missing racial and ethnic information. There were statistically significant differences in various categories among the racial and ethnic groups (Table 1). There were significant differences in marital status between various racial and ethnic groups, with the highest marriage rate for mothers in the Asian group (136 [87.7%]) and lowest for the non-Hispanic Black group (349 [27.0%]) (*P* < .001). The mean body mass index (BMI; calculated as weight in kilograms divided by height in meters squared) at delivery was highest for mothers in the non-Hispanic Black (33.4; 95% CI, 29.4-38.3) and Hispanic (33.1; 95% CI, 29.0-37.0) groups (*P* < .001). Group B streptococcus (GBS) status and cesarean delivery rates were highest for Black (GBS, 206 individuals [36.5%]; *P* = .02; cesarean delivery, 525 individuals [40.7%]) and Hispanic (GBS, 48 individuals [33.1%]; cesarean delivery, 126 individuals [40.0%]) individuals (both *P* < .001). Preterm delivery and preeclampsia rates were highest for mothers in the Black group (preterm delivery, 54 individuals [11.4%]; preeclampsia, 54 individuals [17.1%]; *P* < .001). Gestational diabetes (42 individuals [27.1%]; *P* < .001) and intrauterine growth restriction (15 individuals [9.7%]; *P* = .02) were highest for mothers in the Asian group, and category 2 fetal heart tracing (defined by National Institute of Child Health & Human development criteria) was highest for Asian (34 individuals [21.9%]) and Black (283 individuals [21.9%]; *P* = .02) mothers. Mean neonatal and placental weights were lowest for mothers in the Asian group (neonatal weight, 3110.0; 95% CI, 2815.0-3420.0; *P* < .001; placental weight, 440.0 g; 95% CI, 384.0-529.5 g; *P* = .022) (Table 1, Table 2). Maternal vascular malperfusion of placenta and decidual vasculopathy were highest for Black mothers (mixed type, 112 individuals [8.7%]; *P* < .001; mural hypertrophy, 122 [9.5%]; *P* = .02). Hispanic and non-Hispanic White mothers showed the highest rate of fetal vascular malperfusion (Hispanic, 43 individuals [13.7%]; White, 226 individuals [13.6%]; *P* = .01). Hispanic mothers showed the highest rate of maternal inflammatory response (acute chorioamnionitis, deciduitis, and villitis) (eg, acute chorioamnionitis, 130 individuals [41.3%]; *P* = .02). The meconium stain of fetal membranes rate was found highest in non-Hispanic White mothers (555 individuals [33.4%]).

**Table 1.** Differences in Clinical and Neonatal Features in Singleton Pregnancy by Race and Ethnicity

Characteristics	Individuals, No. (%)					*P* value
	Asian (n = 155)	Black (non-Hispanic) (n = 1291)	Hispanic (n = 315)	White (non-Hispanic) (n = 1662)	Unknown (n = 301)	
Maternal age, mean (95% CI), y	32.0 (29.0-36.0)	31.0 (26.0-36.0)	31.0 (27.0-35.0)	33.0 (28.0-36.0)	31.0 (26.0-35.0)	NA
Marital status						
Single	15 (9.7)	759 (58.8)	143 (45.4)	216 (13.0)	94 (31.2)	<.001
Married	136 (87.7)	349 (27.0)	118 (37.5)	1362 (81.9)	184 (61.1)	
Life partner	3 (1.9)	174 (13.5)	52 (16.5)	69 (4.2)	14 (4.7)	
Other/unknown/declined	0	1 (0.1)	0	2 (0.1)	5 (1.7)	
BMI at delivery, mean (95% CI)	28.9 (25.9-31.4)	33.4 (29.4-38.3)	33.1 (29.0-37.0)	29.3 (26.2-33.4)	29.4 (26.7-33.9)	<.001
GBS status	18 (23.1)	206 (36.5)	48 (33.1)	248 (29.4)	32 (28.8)	.02
SARS-CoV2-positivity	8 (5.2)	64 (5.0)	19 (6.0)	89 (5.4)	19 (6.3)	.87
Gestational age, mean (95% CI), wk	39.0 (38.0-40.0)	39.0 (37.0-40.0)	39.0 (38.0-40.0)	40.0 (39.0-40.0)	39.0 (37.0-40.0)	NA
Delivery mode						
Cesarean	59 (38.1)	525 (40.7)	126 (40.0)	496 (29.8)	107 (35.5)	<.001
Vaginal	96 (61.9)	766 (59.3)	189 (60.0)	1166 (70.2)	194 (64.5)	
Preterm delivery^a^	6 (3.9)	194 (15.0)	36 (11.4)	128 (7.7)	52 (17.3)	<.001
Preeclampsia/PIH	18 (11.6)	307 (23.8)	54 (17.1)	196 (11.8)	39 (13.0)	<.001
GDM2	42 (27.1)	128 (9.9)	53 (16.8)	188 (11.3)	41 (13.6)	<.001
Category 2 heart tracing	34 (21.9)	283 (21.9)	53 (16.8)	295 (17.7)	48 (15.9)	.02
Oligohydramnios	6 (3.9)	25 (1.9)	7 (2.2)	42 (2.5)	7 (2.3)	.59
IUGR	15 (9.7)	64 (5.0)	14 (4.4)	80 (4.8)	7 (2.3)	.02
IUFD	0	24 (1.9)	3 (1.0)	15 (0.9)	6 (2.0)	.07
Neonatal sex						
Female	85 (54.8)	640 (49.6)	154 (48.9)	812 (48.9)	141 (46.8)	.81
Male	70 (45.2)	645 (50.0)	160 (50.8)	845 (50.8)	158 (52.5)	
Neonatal weight, mean (95% CI), g	3110.0 (2815.0-3420.0)	3140.0 (2730.0-3490.0)	3220.0 (2850.0-3550.0)	3330.0 (2960.0-3650.0)	3190.0 (2735.0-3525.0)	<.001
Birth length, mean (95% CI), cm	50.0 (48.0-51.0)	50.0 (48.0-51.5)	50.0 (48.0-51.5)	50.5 (49.0-52.0)	50.0 (48.0-52.0)	NA
Head circumference, mean (95% CI), cm	34.0 (32.5-34.5)	33.5 (32.5-34.5)	33.5 (32.5-35.0)	34.0 (33.0-35.0)	34.0 (33.0-35.0)	NA
Umbilical cord length, mean (95% CI), cm	33.0 (25.5-42.0)	35.0 (27.0-42.0)	35.0 (27.0-43.0)	35.0 (27.0-42.0)	34.0 (25.0-43.0)	.47

Abbreviations: BMI, body mass index (calculated as weight in kilograms divided by height in meters squared); GBS, group B streptococcus; GDM2, gestational diabetes; IUFD, intrauterine fetal demise; IUGR, intrauterine growth restriction; NA, not applicable; PIH, pregnancy-induced hypertension.

^a^ Defined as before 37 weeks.

**Table 2.** Racial and Ethnic Differences in Placental Pathology

Characteristic	Mean (95% CI)					*P* value
	Asian (n = 155)	Black (n = 1291)	Hispanic (n = 315)	White (n = 1662)	Unknown (n = 301)	
Placental weight, g	440.0 (384.0-529.5)	452.0 (381.5-526.5)	461.0 (392.5-532.0)	459.0 (396.0-534.0)	434.0 (381.0-528.0)	.02
Fetal/placental weight ratio	6.9 (6.2-7.8)	6.8 (6.0-7.6)	6.9 (6.2-7.7)	7.1 (6.4-7.9)	7.0 (6.0-7.8)	<.001
Maternal vascular malperfusion, No. (%)						
Decidual vasculopathy						
Classic type	45 (29.0)	354 (27.4)	65 (20.6)	440 (26.5)	71 (23.6)	.10
Mixed type	4 (2.6)	112 (8.7)	15 (4.8)	66 (4.0)	16 (5.3)	<.001
Mural hypertrophy	14 (9.0)	122 (9.5)	28 (8.9)	104 (6.3)	20 (6.6)	.02
Infarctions	7 (4.5)	109 (8.4)	24 (7.6)	104 (6.3)	28 (9.3)	.07
Thrombosis	34 (21.9)	263 (20.4)	51 (16.2)	355 (21.4)	60 (19.9)	.33
Abruption	4 (2.6)	26 (2.0)	9 (2.9)	22 (1.3)	6 (2.0)	.28
Fetal vascular malperfusion (avascular villi), No. (%)	18 (11.6)	124 (9.6)	43 (13.7)	226 (13.6)	27 (9.0)	.01
Inflammatory/infectious						
MIR						
Acute chorioamnionitis	60 (38.7)	424 (32.8)	130 (41.3)	541 (32.6)	110 (36.5)	.02
Chronic deciduitis	47 (30.3)	265 (20.5)	98 (31.1)	425 (25.6)	81 (26.9)	<.001
Chronic villitis	26 (16.8)	179 (13.9)	77 (24.4)	392 (23.6)	53 (17.6)	<.001
FIR (funisitis/fetal vasculitis)	26 (16.8)	178 (13.8)	49 (15.6)	200 (12.0)	48 (15.9)	.12
Meconium stain	40 (25.8)	305 (23.6)	75 (23.8)	555 (33.4)	89 (29.6)	<.001
MPFD/MFI	5 (3.2)	40 (3.1)	10 (3.2)	52 (3.1)	6 (2.0)	.88
Laboratory and other test results						
WBC (x1000), /μL	9.8 (8.2-12.1)	9.0 (7.4-10.8)	9.9 (8.4-12.0)	10.4 (8.7-12.4)	10.3 (8.6-12.5)	NA
Neutrophil differential, %	73.5 (69.2-78.4)	70.3 (65.0-75.3)	73.0 (67.4-76.9)	73.7 (69.4-78.4)	72.7 (68.2-78.4)	NA
Lymphocyte differential, %	17.1 (13.1-21.1)	19.2 (15.1-23.9)	17.9 (14.6-23.0)	17.3 (13.6-21.1)	17.9 (13.6-22.1)	NA
Body temperature, °C	36.7 (36.5-36.9)	36.7 (36.5-37.0)	36.7 (36.5-37.0)	36.7 (36.5-37.0)	36.7 (36.5-37.0)	.67
BMI at delivery	28.9 (25.9-31.4)	33.4 (29.4-38.3)	33.1 (29.0-37.0)	29.3 (26.2-33.4)	29.4 (26.7-33.9)	<.001
Blood pressure						
Systolic	122.0 (115.0-128.0)	129.0 (120.0-140.0)	124.0 (116.0-133.0)	125.0 (117.0-134.0)	126.0 (115.5-135.0)	NA
Diastolic	77.0 (70.0-84.5)	79.0 (71.0-86.0)	77.0 (70.0-83.0)	77.0 (70.0-84.0)	78.0 (71.5-84.0)	NA

Abbreviations: BMI, body mass index (calculated as weight in kilograms divided by height in meters squared); FIR, fetal inflammatory response; MIR, maternal inflammatory response; MPFD/MFI, massive perivillous fibrinoid deposit/maternal floor infarction; WBC, white blood cells.

SI conversion factor: To convert white blood cell count to × 10^9^/L, multiply to 0.001.

## Discussion

Our data showed statistically significant differences of various pregnancy complications and major placental pathology categories among racial or ethnic minority populations. To our surprise, Asian mothers showed significantly higher rates of gestational diabetes and intrauterine growth restriction in our data, and Hispanic mothers showed the highest rates of fetal vascular malperfusion and maternal inflammatory responses, including acute chorioamnionitis, deciduitis, and chronic villitis.^6^ Our data included all the racial and ethnic groups with available data in a racially and ethnically diverse urban community in New York City, and demonstrated the importance of including all racial and ethnic minority groups in analyses when disparities in health outcomes connected to race and/or ethnicity are studied. The underlying etiology of these disparities is multifactorial, and the risk mitigation strategy for pregnant women should be community-centered and risk-targeted, as the risks for different racial and ethnic groups vary significantly.

A limitation in our data was the underrepresentation of Asian and Hispanic women in comparison with non-Hispanic Black and non-Hispanic White women. The socioeconomic status of the patient population was not included in our data, although our local hospital serves most local residents in the neighborhood with assumption of similar socioeconomic status and access to health care available in the city.

Our data showed statistically significant disparities not only of pregnancy complications but also several major placental pathology categories based on race and ethnicity. This study also showed the importance of including all racial and ethnic minority groups when studies of race and ethnicity health outcomes are designed.

## Notes

Supplement.
eMethods.
