# Supplementary material for: Differences in Prevalence of Pregnancy Complications and Placental Pathology by Race and Ethnicity in a New York Community Hospital
Source: JAMA Netw Open. 2022 May 5;5(5):e2210719. doi: 10.1001/jamanetworkopen.2022.10719 (PMC9073565; doi:10.1001/jamanetworkopen.2022.10719)
Supplement: Supplement. — eMethods. [file jamanetwopen-e2210719-s001.pdf]

Supplemental Online Content

Zhang P, Dygulski S, Al-Sayyed F, Dygulska B, Lederman S. Differences in prevalence of pregnancy complications and placental pathology by race and ethnicity in a New York community hospital. *JAMA Netw Open.* 2022;5(5):e2210719. doi:10.1001/jamanetworkopen.2022.10719

eMethods.

This supplemental material has been provided by the authors to give readers additional information about their work.

© 2022 Zhang P et al. *JAMA Network Open.*

eMethods.

The study is exempt from Institutional Review Board (IRB) approval according to section 46.101(b) of 45CFR 46 which states that research involving the study of existing pathological and diagnostic specimens in such a manner that subjects cannot be identified is exempt from the Department of Health and Human Services Protection of Human Research Subjects. It was performed in accordance with STROBE guideline in reporting for cohort study. Placental examination in our institution is criteria-based using the guideline from the College of American Pathologists, and the placentas submitted for pathology examination for a variety of clinical indications in March 2020 and November 2021 were included in the study. Routine paraffin-embedded tissues and Hematoxylin & Eosin (H&E) stained slides were examined by light microscopy using the Amsterdam criteria for placental examination. The placental pathology data were entered into Excel spreadsheet (Microsoft Corporation) at the time of pathology examination, and the neonatal birth data including sex, birth weight, birth length and head circumference were subsequently retrieved from the medical records. Maternal racial/ethnic data were retrieved from the medical record according to the Center for Medicare and Medicaid Services (CMS) criteria as Asian, non-Hispanic Black, Hispanic, and non-Hispanic white. The racial/ethnic data also included “unknown”, “others”, or “declined” as one group without further information. Clinical complications and placental pathological findings were recorded as present or absent. Placental inflammatory / infectious conditions were not grade or staged, and all placental inflammatory responses were included. Only singleton placentas from third trimesters were included in the data, and placentas from first and second trimesters were excluded. Lab tests of white blood counts with differentials and blood pressures measurements were from pre-admission test for delivery only, and before or after delivery blood tests and blood pressure measurements were excluded. Statistical analysis was performed

© 2022 Zhang P et al. *JAMA Network Open.*

by using various programs of R-Package including baseline characteristic table and multi-variant ANOVA tests (http://statistics4everyone.blogspot.com/2018/01/fathers-data-visualization.html).

© 2022 Zhang P et al. *JAMA Network Open.*
